# Linking Genotype to Clinical Features in *SMC1A*-Related Phenotypes: From Cornelia de Lange Syndrome to Developmental and Epileptic Encephalopathy, a Comprehensive Review

**DOI:** 10.3390/genes16101196

**Published:** 2025-10-13

**Authors:** Maria Francesca Astorino, Desirèe Speranza, Giovanni Luppino, Maria Angela La Rosa, Silvana Briuglia, Marco Calabrò

**Affiliations:** 1 Department of Biomedical and Dental Sciences and Morpho-Functional Imaging—BIOMORF, University of Messina, Via Consolare Valeria 1, 98125 Messina, Italy; mastorino@unime.it (M.F.A.); mcalabro@unime.it (M.C.); 2 Department of Chemical, Biological, Pharmaceutical and Environmental Sciences, University of Messina, Via Consolare Valeria 1, 98125 Messina, Italy; desiree.speranza@gmail.com; 3 Department of Human Pathology of Adulthood and Childhood, University of Messina, Via Consolare Valeria 1, 98125 Messina, Italy; giovilup97@gmail.com; 4 UOSD of Genetics and Pharmacogenetics, “Gaetano Martino” University Hospital, Via Consolare Valeria, 98125 Messina, Italy; maria.larosa@unime.it

**Keywords:** *SMC1A* gene, Cornelia de Lange, developmental and epileptic encephalopathy, X-chromosome inactivation, cohesin complex

## Abstract

Germline mutations in the X-linked cohesin subunit gene *SMC1A* have been increasingly recognized as a cause of developmental and epileptic encephalopathy (DEE); however, the underlying basis of its marked phenotypic heterogeneity remains elusive. In our narrative review, starting from all literature-reported clinical cases of *SMC1A*-related DEE, we propose an integrative framework summarizing all the clinical and genetic features, stratified by mutation type, mosaic fraction, and X-chromosome inactivation (XCI) patterns to provide valuable support for genetic diagnosis and variants, found to date. Also, we discuss how somatic mosaicism and epigenetic variability underlie the clinical diversity of *SMC1A*-associated epilepsy and systematically describe the entire phenotypic spectrum, from early-onset, therapy-resistant seizures to milder intellectual disability profiles. We further examine how *SMC1A* mutations perturb cohesin’s canonical roles in chromatin loop formation and sister-chromatid cohesion, leading to widespread transcriptional dysregulation of neurodevelopmental gene networks. Evidence that XCI skewing can ameliorate or exacerbate neuronal cohesin deficits and, thus modulate seizure threshold, is presented.

## 1. Introduction

The *SMC1A* (structural maintenance of chromosomes 1A) gene, also known as *SMC1L1* (Mendelian inheritance in man MIM #300040) according to the revised Human Genome Organization HUGO Gene Nomenclature Committee (HGNC), is a member of the structural maintenance of chromosome (SMC) family that has a relevant role in DNA transactions. SMCs are implicated in the organization of the mitotic and meiotic macroscale chromosome, maintaining genetic stability during the duplication phases [1]. Of them, the *SMC1A* gene, which maps to Xp11.22 in a region that partially escapes X inactivation [2], is a core element of the cohesin multiprotein complex that participates in chromosomal segregation and gene regulation [3]. Specifically, the cohesin complex is a tripartite complex that mediates the cohesion of sister chromatids after DNA replication to ensure faithful chromosome segregation. Cohesin consists of four subunits, two SMC proteins or cohesion core subunits, SMC1A or SMC1B and SMC3, and two non-SMC subunits, RAD21 and either SA1 or SA2 [4]. SMC1A is linked also to cohesion-associated factors that permit cohesion loading in the chromatin such as nipped-B like protein (NIPBL) [5]. In addition, *SMC1A* also plays a role in the DNA damage repair (DDR) pathway, genome stability maintenance, and 3D genome organization [6,7]. *SMC1A* function in the DDR pathway is regulated by ataxia telangiectasia-mutated (ATM) and ataxia telangiectasia- and Rad3-related (ATR) threonine/serine kinases [8]. Additionally, *SMC1A* is also a component of the recombination protein complex (RC-1) that allows DNA repair [9].

Variants in the *SMC1A* gene have been associated with Cornelia de Lange syndrome (CdLS) (MIM #300590), in resembling the Rett syndrome (RTT) phenotype, intellectual disability, and developmental and epileptic encephalopathy (DEE) [10]. More than 100 *SMC1A* variants have been reported in the Human Genome Mutation Database [11], and several of them, mainly both missense and nonsense mutations, have been associated with CdLS. Variants in the *SMC1A* gene are associated with a relatively mild clinical presentation, characterized by the absence of major structural abnormalities commonly seen in CdLS [12], along with only a slight cognitive impairment [13]. In addition, in patients suffering from SMC1A-related CdLS, clinical manifestations are less marked compared to NIPBL-related CdLS cases, the growth rate is relatively preserved, craniofacial dysmorphisms are milder, and cognitive and adaptive functioning are generally higher in the case of *SMC1A* gene mutations [14,15].

*SMC1A* variants are also detected in patients with encephalopathy with epilepsy who do not resemble CdLS. Epileptic encephalopathy, intellectual disability of different grades, and stereotypic movements have also been reported in several patients with likely RTT due to SMC1A variants [14,16,17].

Notably, *SMC1A* variants are described in young patients with DEE. DEE is characterized by refractory seizures, developmental delay, or intellectual disability, which may be caused by gene mutation, also including the *SMC1A* gene. In addition, heterozygous truncation mutations of the *SMC1A* gene are detected in cases of severe early-onset epilepsy with cluster seizures in females [18,19].

Considering the role of *SMC1A* in DNA repair and genome stability maintenance, *SMC1A* variants have been only recently identified in human cancers, even if the role of these variants in oncogenesis is yet to be fully understood [20].

The aim of the narrative review is to evaluate the genetic and clinical features of the *SMC1A* gene mutation with a comprehensive analysis of the role of *SMC1A* variants in developmental and epileptic encephalopathy.

## 2. Materials and Methods

A rigorous search on PubMed and Google Scholar was performed with the terms genes (“SMC1A” OR “Cornelia de Lange Genes” OR “Developmental and Epileptic Encephalopathy Genes”), molecular pathways (“Cornelia de Lange Syndrome” OR “Developmental and Epileptic Encephalopathy”), and “Clinical” and “Diagnostic Approach” (last search 13 August 2025). All manuscripts including cases with clinical information were reviewed.

Outcome inclusion criteria focused on the influence of mutations of the SMC1A gene in CdLS or DEE. Data on the differences in gene expression or in SMC1A regulation were extracted from various publications.

For intervention, exclusion criteria included any study that did not involve SMC1A molecular pathways.

## 3. Genetic, Epigenetic, and Molecular Mechanisms

### 3.1. Molecular Mechanisms of Disease Pathogenesis

Cohesin is a multi-subunit ring complex built around the SMC ATPases SMC1A and SMC3 together with RAD21 and STAG proteins [21,22]. These components are encoded by the *SMC1A*, SMC3, RAD21, and STAG (STAG1/STAG2) genes. Cohesin and its regulatory factors, including the loader NIPBL–MAU2, the unloader WAPL, and the PDS5A/B proteins and HDAC8, cooperate to perform two canonical cellular functions: (1) topological entrapment of sister chromatids to ensure cohesion during and after DNA replication, and (2) active organization of interphase chromatin by loop extrusion, thereby shaping the three-dimensional genome architecture and influencing transcriptional programs [21,22,23,24].

Table 1 summarizes the biological pathways correlated to with SMC1A mutation.

These processes are essential for the correct distribution of genetic material and the regulation of gene expression. Numerous studies have investigated the roles of cohesion, including the specific requirements of subunits for DDR [30,31,32]. From a genetic perspective, impaired cohesin function can underline chromosomal instability and aneuploidy features that contribute to tumorigenesis and cancer progression [30,31,32]. Pathogenic variations in cohesin-related genes are heterogeneous and include missense substitutions, truncating loss-of-function (LoF) variants, splice-site changes, and copy-number alterations [30,31,32]. The molecular consequence of a given allele, dominant-negative versus haploinsufficiency versus hypomorphic effect, strongly influences the clinical phenotype [30,31,32].

Mutations in cohesin genes produce both classical developmental syndromes, exemplified by CdLS, and more recently recognized neurological presentations, including SMC1A-related developmental encephalopathies and SMC1A-DEE [30,32]. Notably, SMC1A is X-linked and displays sex-dependent phenotypic patterns: LoF *SMC1A* variants are reported predominantly in females, and mosaicism or skewed X-inactivation can modulate expressivity, complicating genotype–phenotype interpretation and genetic counseling [30,31,32].

Both missense and truncating *SMC1A* variants have been reported in patients with early-onset, therapy-refractory epilepsy and severe neurodevelopmental impairment [30]. In many cases genotype–phenotype correlations remain incomplete, with closely located *SMC1A* variants associated either with CdLS-like features or with isolated epileptic encephalopathy [30].

One or more cohesin functions may be compromised by *SMC1A* alterations. Mechanistic consequences include defective sister-chromatid cohesion and chromosome segregation, altered loop extrusion or reduced cohesin residence time on chromatin with consequent dysregulation of enhancer–promoter contacts and transcriptional programs, and sensitization to DNA damage through impaired recruitment or retention of cohesin at double-strand breaks [33,34]. Experimental modeling of CdLS-associated *SMC1A* alleles has demonstrated variable impacts on cohesion, mitotic progression, and DNA damage sensitivity, indicating that discrete molecular defects can underline overlapping clinical presentations [33,34].

Beyond these canonical roles, growing evidence implicates cohesin in additional regulatory processes that were less appreciated a decade ago. These include direct modulation of transcriptional elongation and pause-release of RNA polymerase II at specific loci, contributions to the cellular response to replication stress, and an active role in DNA double-strand break repair by promoting homologous recombination and sister-chromatid template usage [21,22]. Large-scale chromatin conformation studies have further clarified how dynamic cohesin loading and unloading shape loop domain landscapes in a cell-type-specific manner and thereby influence developmental gene regulation [21,22].

A particularly active area of inquiry is the interplay between cohesin-driven loop extrusion and phase-separated transcriptional condensates. Recent conceptual and experimental work has proposed that biomolecular condensates enriched for the mediator, BRD4, transcription factors, and RNA polymerase II can cooperate with or constrain cohesin extrusion, modulating enhancer clustering, and enhancer–promoter communication in a context-dependent manner [35,36]. This emerging intersection may explain how modest alterations in cohesin dynamics produce disproportionately large transcriptional effects during neurodevelopment [35,36].

Cohesin activity is tightly regulated by accessory factors that control loading, stabilization on chromatin, and release. NIPBL (with MAU2) is essential for establishing the loop-extruding activity of cohesin; WAPL promotes cohesin release, and limits loop extension; and PDS5 proteins together with *HDAC8* modulate cohesin residence time, and post-translational modification [37,38]. Genetic or functional perturbation of these regulators alters cohesin distribution, loop length, and transcriptional outputs, and several regulators (notably NIPBL) are themselves disease genes in developmental syndromes [37,38]. Consequently, pathogenic effects attributed to *SMC1A* variants can be modified by the functional state of these partners, complicating simple one-gene/one-phenotype models [37,38].

Early studies, published in 2008 by independent groups [39], elucidated molecular links between cohesin function and developmental regulation. Given that cohesins are also expressed in post-mitotic cells, subsequent investigations explored their role in chromatin architecture. Genome-wide mapping of cohesin-binding sites revealed significant overlap with the CCCTC-binding factor (CTCF), highlighting a role for cohesin in transcriptional insulation and long-range regulatory interactions [40].

Being a core element of the cohesin complex, correct SMC1A functioning is pivotal for the physiological role for the SMC1A complex [41]. Any significant alteration of *SMC1A* structure or function due to mutations may profoundly impair the cohesin complex and cause several pathological consequences [41]. Indeed, *SMC1A* mutations disrupt interconnected pathways crucial for genome stability, cell division, DNA repair, epigenetic regulation, and neural development. These alterations can lead to chromosome segregation errors, aneuploidy, and increased genomic instability [26]. Also, alterations reduce efficiency of homologous recombination and other repair pathways, raising mutation rates and cancer risk [26], and perturb chromatin looping, producing widespread transcriptional dysregulation that disproportionately affects developmental and neural genes [8]. Clinically, these molecular defects manifest as disorders such as CdLS, characterized by growth delay, facial dysmorphism, limb anomalies, cognitive impairment [25], and, in some females, early-onset drug-resistant epilepsy and Rett-like encephalopathy, because of X-linked dosage sensitivity and disrupted neural circuitry [30]. In cancer, aberrant *SMC1A* expression or function can contribute to tumor proliferation, cell-cycle dysregulation, and apoptotic resistance, making cohesin a potential therapeutic target [42].

Functionally, *SMC1A* regulates clusters of developmental genes such as *HOX* and *PCDHB* that are important for neurogenesis and neuronal identity [25]. Pathogenic *SMC1A* variants also perturb transcriptional repressors and chromatin insulators, including CTCF, leading to epigenetic instability in a cell-type-specific manner and disrupting genes involved in neuronal differentiation and synaptic signaling [8,43]. CdLS patient-derived cell lines with *NIPBL* and *SMC1A* mutations exhibit altered expression of numerous genes [33,44,45]. Mutations in cohesin subunits are often missense changes or small in-frame deletions and are typically associated with milder forms of the disorder [33].

### 3.2. SMC1A Mutational Spectrum: Genotype–Phenotype Correlations and Clinical Implications

*SMC1A* is generally associated with CdLS and epileptic encephalopathy due to germline mutations. According to reported cases of CdLS children with apparently unaffected parents [46], it has been hypothesized that it could be the results of germline mutations, conventionally involved in gametocytes during oogenesis or spermatogenesis in meiosis [47]. These mutations include missense variants, small in-frame deletions, and, more recently, truncating mutations [48]. Unlike classical CdLS caused by *NIPBL* mutations, SMC1A-related CdLS phenotypes are often milder, lacking major limb anomalies and exhibiting more subtle facial dysmorphism and higher cognitive function [14]. Particularly, truncating variants have been linked to early-onset, drug-resistant epilepsy in females, consistent with X-linked dominant inheritance patterns [14]. Germline mutations in *SMC1A* cause X-linked neurodevelopmental syndromes, most notably a milder variant of CdLS and epileptic encephalopathies in females [14]. From reported cases, different types of germline mutations have been observed:

- Missense and in-frame deletions, typically associated with classic or mild CdLS. They preserve the reading frame and allow partial protein function. Phenotypes include minor facial dysmorphisms and some developmental delay, but absence of major limb defects [14].
- Nonsense and splice-site mutations. These often result in more severe neurological presentations like early-onset epileptic encephalopathy, with seizure onset before age 1 and features resembling the RTT (e.g., stereotypies, regression, profound intellectual disability) [19,49]. Notably, nonsense mutations cluster in regions crucial for cohesin function and have been shown to cause mRNA instability or altered splicing, resulting in loss of function [50].

Additionally, since *SMC1A* is located on the X chromosome and partially escapes X-inactivation, a possible influence of this process has been hypothesized in females, as it will be better described in the next section. Interestingly, most reported pathogenic germline mutations are seen in females [30,51]. These mutations have a functional impact on the role of *SMC1A*. Some mutations lead to aberrant splicing (e.g., intron retention), mRNA decay, and reduced *SMC1A* transcript levels, suggesting haploinsufficiency as a mechanism [49]. Also, the phenotype appears to depend on residual cohesin function. Current evidence suggests that the type and position of *SMC1A* mutations determine clinical outcome. Missense and in-frame deletions often correlate with CdLS-like phenotypes, while truncating mutations, especially those disrupting splicing or critical domains, are enriched in severe epileptic encephalopathies [51].

Although rare, somatic mutations in *SMC1A* have been identified in several cancers, implying a potential role in oncogenesis through impaired chromosome segregation and genome instability. Mostly these are mutations, missense, or small in-frame deletions that preserve the open reading frame. The presence of these alterations suggests the tumor requires a partially functional cohesin complex to survive and proliferate [25]. While no definitive mutational hotspots have been identified and variants are scattered across the gene, it was suggested that they may cluster in functionally significant domains [18].

### 3.3. X-Chromosome Inactivation Skewing and Other Epigenetic Modifiers

In females, one of the two X chromosomes is randomly inactivated in each somatic cell early in embryonic development, a process known as X-chromosome inactivation (XCI). This is crucial for dosage compensation between XX females and XY males. XCI is regulated by epigenetic mechanisms involving X-inactivation-specific transcript XIST RNA, DNA methylation, histone modifications, and chromatin remodeling [52].

Unlike many X-linked genes, *SMC1A* escapes XCI in humans, meaning both alleles are at least partially expressed in females [30,48]. However, the level of escape is incomplete and tissue-specific, which means that the impact of a mutation can vary depending on the degree of XCI and tissue context [30,48].

**Figure 1:**
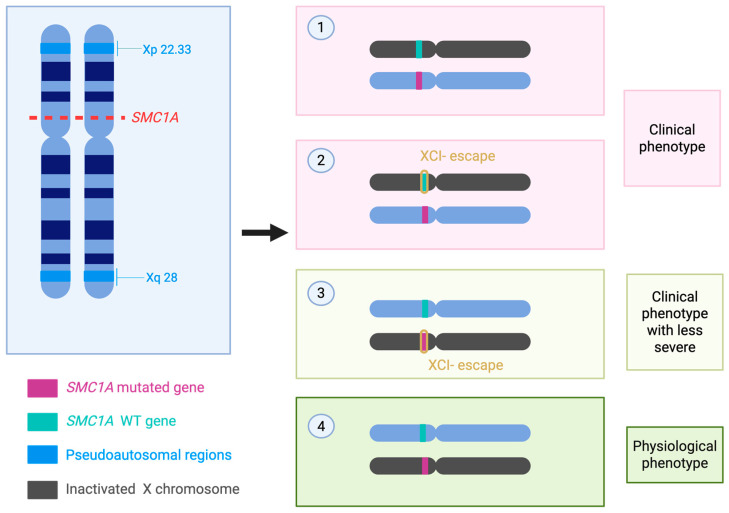
Schematic overview of the impact of *SMC1A* mutations and X-chromosome inactivation (XCI) escape on phenotypic expression. Mutant alleles may partially escape silencing, leading to variable gene dosage. This mechanism contributes to the wide clinical spectrum observed in affected individuals, ranging from physiological phenotypes—defined here as the absence of clinically detectable manifestations, due to sufficient dosage compensation—to milder clinical presentations and severe clinical phenotypes [30]. In the figure we illustrate the four possible events (1) WT *SMC1A*-carrying chromosome is silenced, while Mutated *SMC1A*-carrying chromosome is transcriptionally active. It leads to the production of the mutated protein and thus leads to the pathological phenotype. (2) WT *SMC1A*-carrying chromosome is silenced but SMC1A loci partially escapes X inactivation, while Mutated SMC1A-carrying chromosome is transcriptionally active. It leads to the production of both the mutated and WT (to a less extent) protein and thus leads to the pathological phenotype. (3) WT *SMC1A*-carrying chromosome is transcriptionally active, while the Mutated SMC1A-carrying chromosome is silenced with SMC1A loci partially escaping X inactivation. It leads to the production of both the WT and, to a lesser extent, the mutated protein. Being the WT SMC1A the protein mainly expresses, the phenotype is less severe, even though is still pathological. (4) the WT *SMC1A*-carrying chromosome is transcriptionally active, while the Mutated *SMC1A*-carrying chromosome is silenced. The complete inactivation of the Mutated *SMC1A*-carrying chromosome leads to a physiological phenotype. WT: wild type.

Skewed XCI occurs when one X chromosome is preferentially inactivated over the other [52]. This phenomenon can significantly alter the phenotypic impact of SMC1A mutations in females. If the mutated X chromosome is preferentially inactivated, the functional wild-type allele may dominate, resulting in a milder phenotype [53]. Conversely, if the wild-type X chromosome is preferentially inactivated, mutant *SMC1A* expression predominates, often leading to a more severe phenotype [2,30]. The mechanism of SMC1A’s partial escape from XCI and the associated phenotype are representated in Figure 1.

Studies show that XCI skewing correlates with clinical severity in female patients with early-onset epileptic encephalopathy due to *SMC1A* mutations [18]. Some females with the same mutation exhibit vastly different phenotypes, which supports the notion that XCI skewing is a critical modulator of disease expression [18,30]. Moreover, SMC1A’s partial escape from XCI introduces additional complexity: even when XCI is skewed, incomplete silencing of the mutant allele may permit residual RNA/protein expression from the inactivated X chromosome, which could contribute to pathogenic effects [18,30].

**Figure 2:**
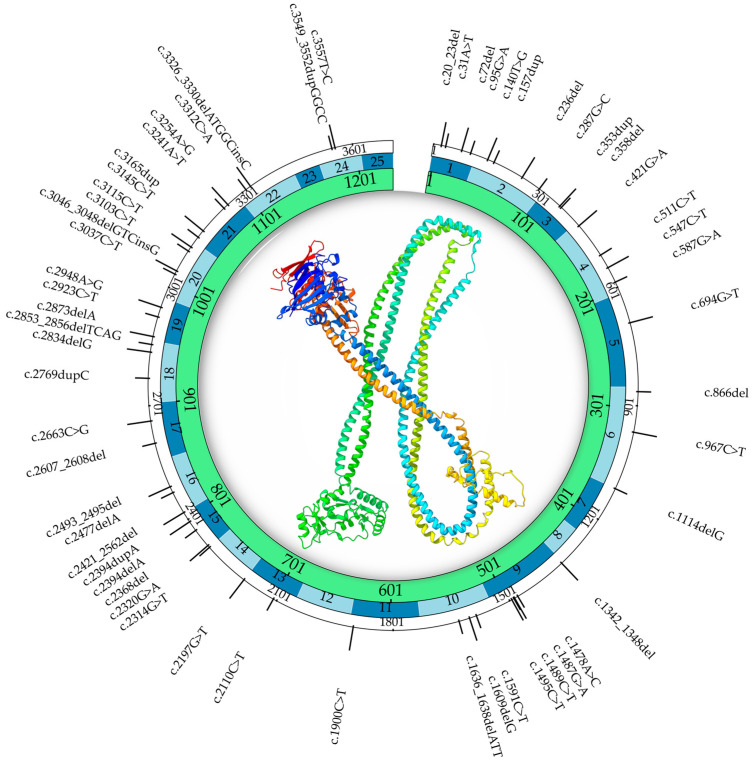
In Figure 2. We report the discussed variants and the positions relative to the CCDS of the *SMC1A* gene. From the outside to the inside, the first ring indicates the nucleotide positions, the second ring indicates the exons, and the third ring indicates the amino acid positions. At the center, we report the 3D structure of *SMC1A* encoded protein. CCDS data were retrieved from the NCBI database, CCDS ID: CCDS14352.1. The 3D structure was based on the crystallographic information of the cohesin complex from UniProt, PDB ID: 6WG3. Only chain A, which represents the SMC1A protein, is reported in the figure. The PDB file was opened and modified with the UCSF ChimeraX software version 1.10.1 [55].

Beyond XCI, other epigenetic modifiers—such as histone acetylation, methylation, and chromatin accessibility—can influence *SMC1A* expression and cohesin function. These modifiers may interact with the mutational background to shape variable outcomes. Epigenetic differences between tissues (e.g., brain vs. blood) likely explain why some individuals with the same *SMC1A* mutation suffer from severe epilepsy while others only show mild cognitive impairment [48]. These findings align with the broader understanding that epigenetic plasticity contributes to phenotypic heterogeneity, especially in X-linked developmental disorders. Table 2 summarizes the clinical variants and phenotypes collected from three case series, while Figure 2 reports the variants and their position in the Certified Clinical Documentation Specialist (CCDS) of the *SMC1A* gene.

## 4. Clinical Phenotypes of SMC1A-Related Disorders

SMC1A-related disorders encompass a heterogeneous clinical spectrum, ranging from non-classical or attenuated CdLS to DEE and other neurodevelopmental phenotypes.

Genotype–phenotype correlations indicate that the molecular nature of the genetic variant (e.g., missense, in-frame, or LoF) is a major determinant [56]. Missense or small in-frame variants are typically associated with milder phenotypes—often within the CdLS spectrum—characterized by subtle craniofacial features, mild growth restriction, and moderate intellectual disability. Conversely, loss-of-function (LoF) variants include nonsense [56]. Nevertheless, the variant type alone does not fully explain the observed variability. Another factor that can substantially influence phenotypic severity is related to XCI. Since *SMC1A* resides on the X chromosome, the relative expression of wild-type and mutant alleles may alter the symptomatology in females.

### 4.1. X-Chromosome Inactivation and Dosage Effects

Skewed XCI may influence SMC1A-related phenotypes, when alterations are present in heterozygosis. When XCI is skewed toward the wild-type allele, functional SMC1A dosage may be preserved, leading to attenuated phenotypes even if a pathogenic variant is present. In contrast, skewing toward the mutant allele or a random XCI pattern can produce a higher proportion of cells with reduced functional SMC1A, driving more severe neurological manifestations, including early-onset, treatment-resistant epilepsy. Considering this, the proportion of cells expressing the mutant versus wild-type allele is critical in determining clinical outcome. This mechanism accounts for some of the variability observed even among individuals with the same variant. Moreover, cellular mosaicism in females may lead to cellular interference, where interactions between wild-type-expressing and mutant-expressing cells generate emergent tissue-level effects, a phenomenon also proposed in other X-linked epilepsies such as the *PCDH19* clustering epilepsy [57]. *SMC1A* variants generally cause more consistent and severe phenotypes, as XCI does not occur to buffer the dosage effect.

Evidence from recent large-scale cohorts supports the clinical relevance of this model. Barañano et al. [10] reported that truncating *SMC1A* variants were frequently associated with skewed XCI, often correlating with severe DEE, whereas missense variants more often showed random XCI and a broader phenotypic range [19], confirming a predominance of LoF alleles (82.1%). These findings have been observed in a multinational cohort of 35 females, with early seizure onset, high drug resistance, and frequent neurodevelopmental regression [19]. Similarly, Fateh et al. described a patient carrying a novel missense variant who, despite profound developmental delay, exhibited no seizures—underscoring the interplay between variant type, XCI pattern, and clinical presentation [58].

Adding to the complexity, *SMC1A* is among the minority of X-linked genes that may escape complete inactivation, leading to biallelic expression in some tissues [44], thus further increasing phenotypic variance.

### 4.2. Cdls vs. SMC1A-DEE: Clinical and Molecular Distinctions

As previously introduced, SMC1A-related disorders exhibit a notable variance spanning from mild CdLS phenotypes to severe DEE cases. While both conditions share a molecular basis in cohesin complex dysfunction, their clinical presentations are distinct.

Notably, while the phenotype of SMC1A-DEE is clearly different from that of CdLS, it bears a strong resemblance to the MECP2-linked RTT, characterized by early-onset, intractable epilepsy and severe-to-profound intellectual disability. While no direct correlation between *SMC1A* and RTT has been described in the literature, variants in other chromatin regulators, such as *HDAC8*, *SATB2*, and *HNRNPU*, have been associated with RTT-like phenotypes [59]. This evidence underscores the dosage-sensitive role of chromatin-associated proteins in early brain development.

Table 3 summarizes the core phenotypic differences between SMC1A-related CdLS and SMC1A-DEE, highlighting the roles of variant type, XCI pattern, and dosage effects in shaping clinical expression.

The etiology of SMC1A-DEE remains poorly understood, partly due to the limited number of reported cases and the challenges in obtaining brain tissue for direct study. Nevertheless, genotype–phenotype correlations highlighted several variants within SMC1A that are correlated with this pathology, as will be better described in the next section. Literature data also suggested that an X chromosome-specific dosage effect, modulated by XCI, plays a major role in determining disease severity [61]. In unaffected females, wild-type alleles are expressed from both the active (Xa) and inactive (Xi) X chromosomes. In SMC1A-DEE, the impact depends on which X carries the loss-of-function variant and how XCI is patterned. Skewing toward the mutant Xi allows most cells to express the WT allele from Xa, resulting in a modest dosage reduction and milder phenotype. In contrast, skewing toward the mutant Xa produces the largest functional loss and the most severe manifestations. Random XCI leads to a mosaic pattern of WT and mutant allele expression, potentially producing an intermediate phenotype. Such mosaicism may also cause cellular interference, a mechanism also proposed for *PCDH19* clustering epilepsy [57], another disorder with drug-resistant seizures and seizure clustering [18].

### 4.3. Case Series

In the Bozarth series [30], one patient exhibited skewed XCI toward the mutant Xi, resulting in a comparatively milder phenotype than those with random XCI—likely reflecting partial preservation of SMC1A dosage. Additional contributions from genes that escape XCI and are expressed from the Xi may also influence sex-specific differences in brain development and phenotypes associated with X-chromosome aneuploidy. Interestingly, the murine ortholog of SMC1A does not escape XCI in the brain [62], suggesting that mouse models may fail to capture the dosage effects observed in humans. For this reason, In vitro neuronal models derived from patient-specific induced pluripotent stem cells (iPSCs) carrying SMC1A-DEE variants on either the Xa or Xi may provide a more faithful platform for functional studies.

Gibellato [19] and colleagues conducted a multicentre study of 35 female patients with SMC1A-related developmental and DEE from 13 countries [19]. The median age at enrollment was 9.5 years (range, 6 months–34 years). No parental consanguinity was reported, and all pregnancies were spontaneous. Prenatal complications included preeclampsia (5.9%) and intrauterine growth restriction (5.7%). Fetal malformations were detected in 14.3% of cases, most commonly holoprosencephaly and arachnoid cysts. Vaginal delivery occurred in 62.9% of cases, with a mean gestational age of 39 weeks. Most patients had normal anthropometric measures at birth; however, 11.4% were small for gestational age in weight, and 8.6% had microcephaly. Neonatal complications occurred in 51.4% of patients, and 34.3% required neonatal intensive care unit (NICU) admission. Major congenital malformations were present in 40% of cases, affecting the cardiovascular system (20%), central nervous system (CNS) (20%), limbs, urinary tract, and genitalia. Postnatal growth was impaired in ~43% of cases, with persistent underweight or microcephaly. Neurodevelopmental milestones were globally delayed. Independent sitting was achieved by 82.4% at a mean age of 14.5 months, walking by 57.6% at 28.5 months, and first words by 56.7% at 26.9 months, while sentence formation was rare (28.6%). Developmental regression affected 51.4%, involving motor, language, and social skills. Behavioral comorbidities occurred in 37.1% cases, including autism spectrum disorder, hyperactivity, and self-injury. Epilepsy onset occurred at a mean age of 11.8 months, most often with generalized seizures (48.6%), frequent status epilepticus (48.6%), and high rates of drug resistance. Only 34.2% cases achieved good seizure control, typically with polytherapy. The ketogenic diet was used in 20% with variable benefit. Gastrointestinal and nutritional issues were common (62.9%), including feeding difficulties, gastroesophageal reflux disease (GERD) (28.6%), and constipation (74.3%). In total, 37.1% required nasogastric tube feeding, and 22.9% had a gastrostomy. Additional medical comorbidities affected 68.6% of patients. Dysmorphic features were observed in 40%, though none met clinical criteria for classical or non-classical CdLS. Genetic testing, predominantly whole-exome sequencing (88.6%), confirmed pathogenic SMC1A variants in all cases, of which 82.1% were loss-of-function alleles, while 17.9% were missense variants.

In 2024, Fateh and colleagues described three clinical cases, including a nine-year-old girl with a pathogenic SMC1A variant [58]. She was born at 36 weeks of gestation to non-consanguineous parents, with a birth weight of 2300 g and a head circumference of 29 cm. Delivery was by cesarean section, with no reported prenatal exposure to medications, tobacco, or alcohol. Dysmorphic features included micrognathia, synophrys, microcephaly, a low anterior hairline, long curly eyelashes, a thin upper lip, a long philtrum, a depressed nasal bridge, and a high-arched palate. Limb anomalies included syndactyly of the second and third toes, small hands with proximally positioned thumbs, and a single palmar crease. Echocardiography revealed a mild ventricular septal defect, while gastroesophageal function was normal. She had no history of seizures, and her electroencephalogram (EEG) was unremarkable. The patient exhibited failure to thrive and global developmental delay, walking independently at the age of four years with occupational therapy, and speaking only single words at age nine. Chromosome analysis showed a normal female karyotype (46,XX). Whole-exome sequencing identified a novel heterozygous SMC1A variant, NM_006306.4: c.2320G > A (p.Asp774Asn), absent in both parents and in population databases (gnomAD, Iranome). The variant was confirmed by Sanger sequencing and classified as likely pathogenic according to the American College of Medical Genetics and Genomics (ACMG) criteria.

In their study, Borck and colleagues [54] described six cases of CdLS associated with SMC1A mutations. Six male patients from four unrelated families were evaluated, and the following variants were identified: p.Asp831Glu/Gln832del, p.Glu493Ala [63], p.Tyr1085Cys, and p.Arg196His. At birth, the mean weight was 2608 g (±601), significantly lower than the population average (*p* < 0.05). The mean birth length was 47.0 cm (±2.8; *p* < 0.01), and the mean occipitofrontal circumference (OFC) was 31.9 cm (±0.5; *p* < 0.05). Postnatal growth parameters remained markedly reduced. The mean height standard deviation score (SDS) was −2.4 (±0.4; *n* = 2), the mean weight SDS was −2.3 (±0.7; *n* = 2), and the mean OFC SDS was −3.4 (±0.9; *n* = 2). Major congenital malformations were observed in 1 of 6 patients (*p* < 0.01), with an average of 1.0 malformation per affected individual. Seizures were reported in 5 of 6 cases (*p* < 0.001). These findings indicate a consistent phenotype characterized by intrauterine and postnatal growth restriction, microcephaly, and a high prevalence of seizures in patients with SMC1L1 missense variants.

## 5. Diagnostic Approaches in Cornelia de Lange Syndrome and SMC1A-Related Developmental and Epileptic Encephalopathy

CdLS is primarily a cohesinopathy caused by pathogenic variants in genes encoding cohesin subunits, regulatory factors, or proteins that modulate cohesin function. The most implicated genes are *NIPBL*, *SMC1A*, *SMC3*, *RAD21*, *HDAC8*, *BRD4*, and *ANKRD11*. De novo *NIPBL* variants account for ~60–65% of clinically ascertained cases while the remaining genes explain a smaller proportion and contribute to phenotypic heterogeneity [64]. International consensus criteria distinguish cardinal and suggestive features to guide clinical recognition. A diagnosis is established by characteristic clinical findings or identification of a pathogenic (or likely pathogenic) heterozygous variant in *NIPBL*, *RAD21*, *SMC3*, or *BRD4*, or a hemizygous pathogenic variant in *HDAC8* or *SMC1A* [64].

For molecular testing, clinician-directed gene-targeted strategies that include multigene panels or serial single-gene analysis are appropriate when CdLS is strongly suspected. Recommended panels should include *NIPBL*, *SMC1A*, *HDAC8*, *SMC3*, *RAD21*, *BRD4,* and genes causing overlapping phenotypes (e.g., AFF4, *ANKRD11*, *CREBBP*, *EP300*), and should incorporate deletion/duplication analysis. Because somatic mosaicism is relatively frequent, NGS methods capable of detecting low-level mosaic variants—ideally applied to uncultured fibroblasts or other mosaic-enriched tissues (e.g., buccal epithelial cells)—are advised. Serial testing—considered in settings where multigene panels are unavailable—is the first step for individuals with high clinical suspicion. It typically begins with *NIPBL* (sequence ± deletion/duplication), followed by *SMC1A* in milder phenotypes, and then *BRD4*, *SMC3*, *RAD21*, and *HDAC8* as indicated. When the phenotype is atypical or CdLS is not initially suspected, comprehensive genomic testing—such as exome or genome sequencing—is the preferred diagnostic route [64].

Molecular diagnosis of SMC1A-related DEE relies primarily on NGS strategies, including targeted epilepsy/CdLS gene panels and trio whole-exome or whole-genome sequencing, which have markedly increased detection of de novo SMC1A loss-of-function variants in females presenting with early-onset, drug-resistant seizures and severe neurodevelopmental impairment [10,65]. Because SMC1A is X-linked and can partially escape X-chromosome inactivation, allele-specific expression and X-inactivation analyses (and interpretation of variant dosage) are often informative when correlating genotype with phenotype [2,30]. Given the documented occurrence of post-zygotic mosaicism and the possibility that causal variants may be absent or at very low allele fractions in peripheral blood, diagnostic workups should include deep sequencing capable of mosaic detection and multi-tissue sampling (uncultured fibroblasts, buccal epithelial cells, or other mosaic-enriched tissues) when clinical suspicion remains high despite a negative blood test [66,67]. Complementary assays—copy-number analysis (array comparative genomic hybridization (CGH), multiplex ligation-dependent probe amplification (MLPA), or deletion/duplication algorithms applied to NGS data)—are recommended to exclude intragenic deletions or duplications not detected by standard sequencing [64]. For variants predicted to affect splicing, RNA-based studies (Reverse Transcription Polymerase Chain Reaction (RT-PCR) from appropriate tissues, RNA-seq, or minigene splicing assays) can demonstrate aberrant transcripts and help reclassify variants of uncertain significance. Functional characterization in cellular models (e.g., patient-derived or gene-edited iPSC-derived neurons) and targeted molecular assays (protein expression, allele-specific expression) are increasingly used to validate pathogenicity and to investigate mechanistic effects on neuronal function, although such assays are generally performed in research settings or specialized diagnostic laboratories [68]. Table 4 summarizes diagnostic techniques for SMC1A in DEE in particular the advantages and limitations in clinical practices.

### 5.1. Current Molecular Strategies

Diagnostic workflows for SMC1A in DEE must combine sensitive sequencing, copy-number interrogation, and strategic tissue selection to maximize detection of both germline and post-zygotic (mosaic) variants. When the clinical picture suggests SMC1A involvement (early-onset, drug-resistant seizures with developmental impairment or CdLS-like features), begin with a clinician-directed multigene NGS panel or trio WES/WGS; panels optimized for epilepsy/CdLS usually afford the best balance of coverage and interpretability, while exome/genome sequencing is preferable for atypical presentations. Multigene panels should explicitly include deletion/duplication analysis and parameters for mosaic calling [64].

Because pathogenic SMC1A variants can be mosaic- and tissue-restricted, peripheral blood alone may be insufficient. Empirical data show that buccal epithelial cells and uncultured skin fibroblasts frequently enrich for mosaic alleles missed in blood; therefore, collect a non-blood specimen (buccal swab or fibroblast biopsy) at the time of initial testing when clinically feasible or pursue reflex testing if blood is negative but suspicion remains high [76]. Multi-tissue sampling increases diagnostic yield and can reveal gonadosomatic mosaicism relevant to recurrence risk [64,76].

Analytical sensitivity depends on sequencing depth and the bioinformatic pipeline. Standard clinical exome/panel pipelines typically detect mosaic variants reliably at moderate variant allele fractions (VAFs)—often ≈5–10%—whereas Sanger sequencing generally fails below ~15–20% VAFs. Detecting VAFs approaching 1% requires targeted ultra-deep amplicon sequencing with robust error-correction and validated limit-of-detection (LLoD). Laboratories should define and report their LLoD and use mosaic-aware variant callers and filtering strategies validated against reference standards. Benchmarking studies recommend selection and parameter optimization specific to anticipated VAF ranges [77].

Complementary assays are essential: CNV analysis (array CGH/MLPA/NGS-based algorithms) to detect intragenic deletions/duplications, RNA studies (RT-PCR, RNA-seq, or minigene assays) for splice variants, and X-inactivation/allele-specific expression tests in females to contextualize variant impact given the X-linkage of the SMC1A gene and potential X-escape. Confirmatory testing of low-level variants should use an orthogonal method (e.g., independent ultra-deep amplicon sequencing). Finally, variant interpretation should follow ACMG-adapted rules for mosaic findings and incorporate phenotypic concordance, tissue distribution, parental testing for recurrence risk, and, when available, functional evidence from RNA or cellular models. Clear laboratory reporting of tissue source, VAF, LLoD, and interpretative caveats is critical for clinical decision-making [66,78]

Multiple studies investigating CdLS and SMC1A-related disorders have demonstrated that a multimodal diagnostic strategy combining conventional and high-resolution molecular methods across different tissues substantially improves variant detection and classification [13,79,80]. Traditional Sanger sequencing and chromosomal microarray analyses remain useful first-line tools but can fail to detect low-level post-zygotic variants or complex rearrangements. In contrast, next-generation sequencing (NGS) approaches, including targeted gene panels, exome/genome sequencing, and ultra-deep amplicon assays, increase sensitivity for single-nucleotide variants and permit mosaic-aware calling when coupled with appropriate bioinformatic pipelines. Complementary techniques such as copy-number interrogation, RNA-based splicing assays, and tissue-specific sampling (e.g., buccal cells, uncultured fibroblasts, bone marrow) further enhance diagnostic yield and enable functional interpretation, while cellular models provide mechanistic validation in selected cases. Collectively, these studies support an integrated, tissue-informed workflow for resolving diagnostically challenging cases.

To address these diverse clinical and economic demands, in their study, Balciuniene et al. [81] developed an exome sequencing (ES)-based test for pediatric epilepsy diagnosis, focusing on an initial analysis of 100 epilepsy genes. The ES-based strategy enables an easy reflex to analysis of the full exome for cases with a negative panel outcome and offers flexibility in modifying the panel’s gene content in response to new gene discoveries or clinical demands [81].

In another study including three patients, prior Sanger sequencing of peripheral blood DNA failed to identify pathogenic variants in any of the five canonical CdLS genes [82]. Subsequent analyses of blood DNA by exome sequencing (patient B) and chromosomal microarray (patients B and C) also did not reveal causative alterations. Consequently, a high-coverage NGS approach was pursued on DNA extracted from bone marrow. For this purpose, a custom AmpliSeq panel (AmpliSeq Designer, Life Technologies, Darmstadt, Germany) was designed for the Ion Torrent PGM platform that incorporated the five established CdLS genes alongside additional candidate loci. The final design was split into two libraries and covered >95% of an approximately 75 kb targeted genomic interval. In summary, these results support a substantial contribution of mosaic *NIPBL* mutations among individuals with classic CdLS phenotypes who were negative based on conventional Sanger sequencing of blood, or even bone marrow, DNA. Given the limited availability of patient fibroblasts in many diagnostic settings, these findings underscore the urgent need to integrate highly sensitive NGS-based methods into routine molecular diagnostics for CdLS. They therefore strongly recommend applying such mosaic-sensitive sequencing approaches for patients with a clinical diagnosis of CdLS who remain mutation-negative following standard sequencing of lymphocyte or bone marrow DNA [82].

### 5.2. Limits

While this review aimed to collect and summarize the available literature on the role of SMC1A, a limitation that should be acknowledged is related to the paucity of published studies, which restricts the evidence base. This inevitably narrows the scope of comparative analyses and limits the generalizability of the conclusions, underscoring the need for caution in interpreting the findings. Nevertheless, it should be noted that the pathologies discussed are rare and relatively understudied, which helps explain the limited amount of evidence available in the literature.

## 6. Conclusions

Variants of the *SMC1A* gene are associated with a broad clinical spectrum ranging from attenuated CdLS to SMC1A-related DEE. This reflects the diverse functions of the cohesin complex, which include sister-chromatid cohesion, damaged DNA repair, 3D chromatin structure, and gene expression control. Missense variants and small in-frame variants tend to have lower severity and are associated with CdLS-like features, while loss-of-function variants (nonsense, frameshift, and splicing-altering variants) are more frequent in individuals with severe neurodevelopmental disorders and early-onset refractory epilepsy. Sequence variants are not the only source of functional alteration in SMC1A. In females, the partial escape of SMC1A from XCI, together with skewed XCI, generates tissue-specific cellular mosaics that alter effective SMC1A dosage and thereby magnify interindividual phenotypic variability. From a diagnostic standpoint, the best results come from sequential multimodal analysis of XCI and allele-specific expression in women, integrated with NGS (targeted panels, exome/genome), detection of mosaic variants and copy number, and RNA-based tests involving splicing defects and XCI/allele expression dynamics. Due to the potentially tissue-limited nature of pathogenic *SMC1A* variants, multitissue sampling (e.g., blood plus buccal epithelium or fibroblasts) is often critical to identify low-level mosaicism, classify variants, and provide accurate counseling. In the clinical setting, supportive care remains the mainstay, with symptomatic treatment focused on seizure control, nutritional management, and multidisciplinary neurological development support. SMC1A disorders remain examples of a X-linked, dose-sensitive paradigm in which a combination of genetic lesions, epigenetic context, and tissue mosaicism converge to influence the phenotype, revealing the importance of personalized molecular diagnostics and precision in care pathways.

## Figures and Tables

**Table 1:** SMCA1 mutation and biological pathways.

Biological Pathway	Effect of SMC1A Mutation	References
Chromatid cohesion	Chromosomal instability and segregation errors	[25]
DNA damage repair (Homologous recombination HR, Non homologous end joining NHEJ)	Reduced repair fidelity, genomic instability	[26]
Gene transcription and chromatin	Misregulation of developmental genes and epigenetic control	[8]
Cell cycle control	G1/S or G2/M arrest, reduced proliferation, increased apoptosis	[27,28,29]
Neural development	Epilepsy, intellectual disability, Rett-like features in females	[30]

**Table 2:** Mutations and associated phenotypes reported in the literature.

#	Nucleotide Changes	Amino Acid Changes	Type	XCI	Age of Seizure Onset	Speech	ID	Walking	MRI
1	c.20_23del	Ile7Argfs * 42	Frameshift	Skewed 81:19	15 mo	None	Yes	No	N/A
2	c.31A > T	Asn11Tyr	Missense	N/A	2.5 mo	N/A	Yes Yes	N/A	Normal
3	c.140T > G	Phe47Cys	Missense	Random 7:26	3 mo	None	Yes Yes	No	N/A
4	c.157dup	Thr53AsnfsX34	Frameshift	N/A	5 mo	N/A	Yes Yes	N/A	Cerebral volume loss
5	c.287G > C	Arg96Pro	Missense	Random 7:26	18 mo	N/A	Yes Yes	N/A	N/A
6	c.421G > A	Glu141Lys	Missense	Highly skewed 100:0	2.5 mo	None	Yes Yes	N/A	N/A
7	c.511C > T	Arg171Ter	Nonsense	Random	4 wk	None	Yes Yes	No	Normal
8	c.615G > A	Glu2055 *	Splice-site	N/A	P3 (18) 4 mo; P12 (22): 13 mo	None	Yes Yes	No	P3: small hemorrhage along with posterior falx and tentorium
9	c.615G + 1G > C	Glu2055 *	Splice-site	Moderately skewed 83:17	1 mo	N/A	Yes Yes	No	N/A
10	c.615 + 5G > A	Glu2055 *	Splice-site	Random	4 mo	None	Yes	No	Normal
11	c.694G > T	Glu232Ter	Nonsense	N/A	4 mo	N/A	Yes	N/A	N/A
12	c.1113 + 1G > A	Gln371fs *	Splice-site	N/A	7 wk	None	Yes	N/A	N/A
13	c.1114delG	Val372Ter	Nonsense	N/A	7 wk	None	Yes	N/A	N/A
14	c.1487G > A	Arg496His	Missense	Skewed in patient P3	35 mo	limited	Yes	Yes	N/A
15	c.1489C > T	Arg497Ter	Nonsense	N/A	4 mo	None	Yes	N/A	N/A
16	c.1495C > T	Arg499Ter	Nonsense	N/A	40 mo	None	Yes	Yes	Microform of HPE, ventricular ectasia
17	c.1591C > T	Gln531Ter	Nonsense	N/A	15 mo	None	Yes	Yes	Normal
18	c.1609delG	Val537Phefs * 42	Frameshift	N/A	5 mo	None	Yes	No	N/A
19	c.1636_1638delATT	Ile546del	In-frame	Moderately skewed 86:14, Mosaic	2 mo	None	Yes	No	N/A
20	c.1900C > T	Gln634Ter	Nonsense	N/A	3 mo	N/A	Yes	N/A	N/A
21	c.1911 + 1G > T	Thr638Valfs * 48	Splice-site	N/A	Neonate	None	Yes	no	Small frontal lobe, thin CC
22	c.2197G > T	Glu733Ter	Nonsense	Random	5 mo	None	Yes	No	Volume loss
23	c.236del	Asn788Lysfs * 10	Frameshift	Random	9 mo	None	Yes	No	Slightly enlarged ventricles, hypoplastic cerebellar vermis
24	c.2394delA	Lys798Asnfs * 31	Frameshift	N/A	N/A	N/A	Yes	N/A	Semi-lobar HPE
25	c.2394dupA	Arg799fs	Frameshift	N/A	4 mo	None	Yes	N/A	N/A
26	c.2421_2562del	Leu808Argfs * 6	Frameshift	Moderately skewed 85:15	2 mo	None	Yes	N/A	Mild periventricular white matter abnormalities
27	c.2477delA	p825fs	Frameshift	N/A	28 mo in P8; <1 mo in P9	P8: None	P9: None	P8: Yes	P9: normal; P8: hemi-lobar HPE
28	c.2663C > G	Arg898Gly	Missense	Skewed; P1/P2	25 mo; P1	None	Yes	P1: Yes; P2: normal until onset	P2: cerebellar atrophy
29	c.2769dupC	Ser924Glnfs * 2	Frameshift	N/A	24 mo	N/A	Yes	N/A	N/A
30	c.2834delG	Gly945Lysfs * 19	Frameshift	N/A	N/A	N/A	Yes	N/A	Semi-lobar HPE
31	c.2853_2856delTCAG	Ser951Argfs * 12	Frameshift	Skewed	4 mo	N/A	severe	no	Mild ventriculomegaly
32	c.2873delA	Gln958Argfs * 6	Frameshift	Random	3 mo	None	Yes	No	N/A
33	c.2923C > T	Arg975Ter	Nonsense		5 mo	Moderate to severe	no	Yes	Normal
34	c.3046_3048delGTCinsG	Val1016Alafs * 28	Frameshift	N/A	Neonate	None	Yes	No	Thin abnormal CC and minimal cerebellar atrophy
35	c.3115C > T	Gln1039Ter	Nonsense	Random 76:24	2 mo	None	Yes	No	N/A
36	c.3145C > T	Arg1049Ter	Nonsense	N/A	5–6 wk	None	Yes	no	Cerebral volume loss
37	c.3241A > T	Ile1081Phe	Missense	N/A	4 yr	None	Yes	Yes	Mildly prominent lateral ventricles
38	c.3285 + 1G > C	p1095	Splice-site	N/A	Not reported	N/A	Yes	N/A	Middle interhemispheric variant, HPE
39	c.3312C > A	Tyr1107Ter	Nonsense	N/A	12 yr	Normal before SE	Yes	Yes	Normal
40	c.3326_3330delATGGCinsC	Asp1109Alafs * 102	Frameshift	N/A	6 mo	None	Yes	no	Small cavum septum vergae
41	c.3549_3552dupGGCC	Ile1185glyfs * 23	Frameshift	Random in P1 (59)-N/A-P3-(22)	17 mo P2-; 16 (59)mo P13-(22)	N/A-Goldstein; P13-Baranano-limited	Yes	N/A-; P13(59)(22)-Yes	P2 (59): mild enlarged extra-axial spaces and slight thinning of CC
42	c.2320G > A	Asp774Asn	Missense	N/A	N/A	delay	Yes	At 4 yr	N/A
43	c.3103C > T	Arg1035Ter	Nonsense						
44	c.1342_1348del	Ser448Lysfs * 6	Frameshift/Truncating						
44	c.967C > T	Gln323Ter	Nonsense						
45	c.2368del	Arg790Glyfs * 8	Frameshift/Truncating						
46	c.866del	Ser289Ter	Nonsense						
47	3428bp Deletion	-	Microdeletion						
48	c.2110C > T	Gln704Ter	Nonsense						
49	c.3557T > C	Val1186Ala	Missense						
50	c.358del	Glu120Asnfs * 2	Frameshift/Truncating						
51	c.72del	Gln25Argfs * 25	Frameshift/Truncating						
52	c.3165dup	Lys1056Insfs * 13	Frameshift/Truncating						
53	c.353dup	Ser118Argfs * 2	Frameshift/Truncating						
54	c.3037C > T	Gln1013Ter	Nonsense						
55	c.2314G > T	Val772Leu	Missense						
56	c.2948A > G	Tyr983Cys	Missense						
57	Ex1 del	-	Microdeletion						
58	c.95G > A	Gly326Glu	Missense						
59	c.2607_2608del	Gln869Hisfs * 17	Frameshift/Truncating						
60	c.547C > T	Gln183Ter	Nonsense						
61	c.2493_2496del	Asp831Glu/Gln832del	Missense/Del In-frame						
62	c.1478A > C	Glu493Ala	Missense						
63	c.3254A > G	Tyr1085Cys	Missense						
64	c.587G > A	Arg196His	Missense						

Table 2 provides a list of variants and related clinic phenotypes. Patients from 1 to 41 were already reported in Bozarth et al. [30] (2023); patients from 42 to 58 were already reported in Gibellato et al. [19] (2024); and patients from 59 to 64 were already reported in Borck et al. [54] (2007). Variants were compared, and duplicated entries were removed. XCI: X-chromosome inactivation; ID: intellectual disability; MRI: magnetic resonance imaging; SE: status epilepticus; HPE: holoprosencephaly; CC: corpus callosum; P(N#): patient numbers from the original clinical reports or cohorts. *: stop-codon. N/A: data not available.

**Table 3:** Clinical DEE phenotypes summary [30,60].

Primary Clinical Presentation	Multisystem Developmental Disorder	Severe Neurodevelopmental Disorder with Epilepsy
**Neurological features**	Mild-to-moderate intellectual disability; variable motor delay	Profound developmental impairment; hypotonia; regression possible
**Seizures**	Rare or absent	Early-onset, often refractory epilepsy
**Neurodevelopmental course**	Stable developmental impairment	Progressive decline related to epileptic activity
**Facial dysmorphism**	Distinctive CdLS facial features (arched eyebrows, long eyelashes, short nose, thin upper lip)	Absent or subtle facial dysmorphism
**Growth parameters**	Prenatal and postnatal growth retardation	Normal growth parameters in many cases
**Other systemic involvement**	Possible limb anomalies, gastrointestinal and cardiac defects	Less frequent systemic malformations
**Genetic cause (SMC1A context)**	Pathogenic variants in SMC1A (often missense or in-frame changes)	Pathogenic variants in SMC1A (often LoF, splice-site, or truncating)
**Onset**	Congenital	Infancy (usually within the first year)

**Table 4:** Diagnostic techniques for SMC1A in DEE.

Method	Advantages	Limitations	Specimen/Practical Notes	References
Multigene Next generation sequencing (NGS) panel (epilepsy/CdLS panels)	High diagnostic yield when phenotype is suggestive; targeted content increases interpretability; panels can combine single-nucleotide variant (SNV) and copy number variation (CNV) detection.	May miss very low-level mosaic variants if sequencing depth is limited; gene content and sensitivity vary between laboratories.	DNA from peripheral blood; collect additional mosaic-enriched tissue (buccal swab, uncultured skin fibroblasts) if mosaicism is suspected. Panels should include validated CNV detection.	[64]
Trio whole-exome sequencing (WES)/whole-genome sequencing (WGS)	Broad coverage for atypical presentations; facilitates de novo variant detection and parental phasing.	Standard WES/WGS pipelines often lack sensitivity for very low variant allele frequency (VAF) mosaicism; cost and analytic complexity are higher.	DNA from proband + parents (trio) recommended. Follow-up targeted testing or deep sequencing may be required for mosaic detection.	[65,69]
Ultra-deep targeted amplicon NGS	Sensitive detection of low VAF mosaic variants (with appropriate error-correction); ideal for confirmatory testing.	Requires locus-specific assay design, rigorous error-correction and validation; not genome-wide.	Multi-tissue sampling recommended (blood, buccal, fibroblasts). Typical target depths ≥ 500–1000× with validated limit of detection.	[70,71]
Copy-number analysis (Array comparative genomic hybridization (a-CGH)/Multiple ligation-dependent probe (MLPA)/NGS-based CNV calling)	Detects intragenic deletions/duplications not identified by SNV calling.	May have reduced sensitivity for low-level mosaic CNVs and cannot detect single-nucleotide variants.	DNA from blood or alternative tissues when mosaicism is suspected. Integrate with sequencing results.	[72,73]
RNA-based assays (Reverse transcription (RT-PCR), RNA-seq, minigene splicing assays)	Provide functional evidence for splice-altering variants and quantify transcript consequences; aid reclassification of variant of uncertain significance (VUS).	Require expression of *SMC1A* in the sampled tissue; patient RNA may be unavailable or low expressing.	Use tissue with relevant expression; if unavailable, deploy minigene constructs or fibroblast RNA where feasible.	[74]
Functional cellular models (Induced pluripotent stem cells iPSC-derived neurons, gene-edited cellular assays)	Directly assess cellular/neuronal consequences of variants and support pathogenicity assignments.	Resource-intensive, time-consuming, and generally confined to research or specialized diagnostic labs.	Derive iPSCs from patient fibroblasts or blood; used for mechanistic studies and advanced validation.	[68,75]
X-inactivation/allele-specific expression assays	Critical for interpreting variant effect in heterozygous females given X-linkage and variable X-escape; refines genotype–phenotype correlation.	Interpretation can be complex due to tissue-specific X-chromosome inactivation (XCI) patterns and mosaicism.	Perform XCI assays on multiple tissues when possible; combine with allele-specific expression and phenotypic data.	[66]

## Data Availability

No new data were created or analyzed in this study.

## Abbreviations

The following abbreviations are used in this manuscript:

ATM	Ataxia telangiectasia-mutated
ATR	Ataxia telangiectasia- and rad3-related
CdLS	Cornelia de Lange syndrome
CTCF	CCCTC-binding factor
DEE	Developmental and epileptic encephalopathy
iPSCs	Induced pluripotent stem cells
LoF	loss of function
NGS	Next-generation sequencing
NIPBL	Nipped-B like protein
RC-1	Recombination protein complex
RT-PCR	Reverse transcription polymerase chain reaction
RTT	Rett syndrome
SMCs	Structural maintenance of chromosomes
SMC1A	Structural maintenance of chromosomes 1A
WES	Whole-exome sequencing
WGS	Whole-genome sequencing
Xa	X active
XCI	X-chromosome inactivation
Xi	X inactive
